# Medicaid Expansion and Survival Outcomes Among Men With Prostate Cancer

**DOI:** 10.7759/cureus.77434

**Published:** 2025-01-14

**Authors:** Oluwasegun A Akinyemi, Mojisola E Fasokun, Eric Hercules, Ayodeji Ikugbayigbe, Eunice Odusanya, Lakin Hatcher, Nadia Hackett, Oluebubechukwu Eze, Lerone Ainsworth, Miriam Micheal, Kakra Hughes, Pamela W Coleman

**Affiliations:** 1 Clive O. Callender Outcomes Research Center, Howard University College of Medicine, Washington, D.C., USA; 2 Department of Epidemiology and Public Health, University of Alabama at Birmingham, Birmingham, USA; 3 Department of Biological Sciences, Eastern Illinois University, Charleston, USA; 4 Department of Surgery, Howard University College of Medicine, Washington, D.C., USA

**Keywords:** affordable care act, cancer-specific survival (css), georgia, kentucky, medicaid expansion, overall survival (os), prostate cancer

## Abstract

Introduction: Prostate cancer stands as one of the most diagnosed malignancies among men worldwide. With the recent expansion of Medicaid under the Affordable Care Act (ACA), millions more Americans now have health insurance coverage, potentially influencing healthcare access and subsequent outcomes for various illnesses, including prostate cancer. Yet, the direct correlation between Medicaid expansion and cancer-specific survival among individuals with prostate cancer remains an area warranting comprehensive exploration.

Objective: This study aims to determine the impact of the implementation of Medicaid expansion on survival outcomes among men with prostate cancer.

Methods: We utilized data from the Surveillance, Epidemiology, and End Results (SEER) registry to determine the causal impact of the implementation of the ACA on outcomes among men with prostate cancer. The study covered the years 2003-2021, divided into pre-ACA (2003-2009) and post-ACA (2015-2021) periods, with a one-year washout (2014-2015) since Medicaid expansion was implemented in 2014 in Kentucky. Using a difference-in-differences (DID) approach, we compared survival among men with prostate cancers from Kentucky to Georgia. We adjusted for patient demographics, income, metropolitan status, disease stage, and treatment modalities.

Results: We analyzed a cohort of 68,222 men with prostate cancer during the study period. Of these, 37,810 (55.4%) were diagnosed in the pre-ACA period, with 70.8% from Georgia and 29.2% from Kentucky. The remaining 30,412 (44.6%) were diagnosed in the post-ACA period, with 72.3% from Georgia and 27.7% from Kentucky. Medicaid expansion in Kentucky was associated with a 16.8% reduction in the hazard of overall death, indicating improved overall survival among eligible individuals. This trend was consistent across different racial and ethnic groups. Specifically, non-Hispanic White men experienced a 16.2% reduction (DID=-16.2%; 95% CI: -31.5% to -0.8%), non-Hispanic Black men had a 17.9% reduction (DID=-17.9%; 95% CI: -34.8% to -0.9%), and Hispanic men saw a 15.9% reduction (DID=-15.9%; 95% CI: -31.3% to -0.5%) in hazard of death among low-income individuals.

Conclusion: Medicaid expansion was associated with a substantive improvement in overall survival among men with prostate cancer in Kentucky compared to non-expansion Georgia.

## Introduction

Outside of skin cancer, prostate cancer is the most common cancer in men in the United States [1]. Approximately 299,010 new cases of prostate cancer are expected in 2024, along with about 35,250 deaths from the disease [2]. Since 2014, the incidence rate has increased by 3% per year overall and 5% per year for advanced-stage prostate cancer [2]. For men diagnosed with prostate cancer, the treatment can range from observation, curative surgery (prostatectomy), targeted radiation therapy, and other treatment modalities directed at advanced cases of the disease [3]. However, what is not often discussed are the factors that determine the treatment of choice [3,4]. Financial toxicity is a term that is used to describe the financial consequences and the potential stress incurred by a disease diagnosis and treatment [5]. In 2020 alone, there was an estimated $22.3 billion spent on prostate cancer care, with the majority of that constituting direct medical expenses [6]. However, the economic impact goes far beyond medical bills. Prostate cancer leads to a very significant loss of earnings as a result of premature mortality and the inability of patients to work during their treatment and recovery process [7].

Apart from the financial impact of prostate cancer, the complex pathogenesis of the disease also involves genetic and environmental factors and is mainly driven by alterations in androgen signaling [8]. There are a multitude of risk factors that contribute to the development of prostate cancer, including age, family history, and race [9]. African American men are at a higher risk of developing prostate cancer and usually tend to present with a more advanced disease at diagnosis compared to their counterparts [10]. It has also been shown that social determinants of health (SDOH) have a significant impact on prostate cancer outcomes [11]. Factors such as socioeconomic status, access to healthcare, and living conditions influence not only the stage at which prostate cancer is diagnosed but also overall survival (OS) rates [11]. Prior data suggests that incidence rates are associated with socioeconomic status [10]. Additionally, lower socioeconomic status is associated with poorer survival rates [10]. In order to reduce disparities in prostate cancer care and improve survival, it is crucial to address the SDOH that may be impacting the outcomes of the disease.

The goal of Medicaid, initially enacted in 1965, was to allow states to receive federal funding to provide health insurance to persons with limited income [12]. This legislation was later expanded and termed the Affordable Care Act (ACA), passed in 2010 and fully implemented in 2014 [13]. This legislation addressed Americans without health insurance facing systemic health inequalities [13]. This act is the most significant expansion of coverage in the US healthcare system following the creation of Medicare and Medicaid in 1965 [14]. Before the ACA, there were limitations to Medicaid eligibility on the state level, which covered only the poorest in specific categories (i.e., disabled people, pregnant women, and children) [14]. In contrast, with the ACA, citizens with income at or below 138% of the federal poverty line qualify for aid [13]. Due to the ACA, the number of uninsured individuals declined significantly from 2013 to 2022, from approximately 45.2 million to 26.4 million [5]. The overall implementation of the ACA during 2013-2022 has led to an overall 45% increase in Americans with health insurance coverage [15].

When looking specifically at the effect of the ACA on patients newly diagnosed with breast, colorectal, and lung cancer from 2012 to 2015, there have been an increase in earlier stages of diagnosis and a lower hazard of mortality among patients [16]. A study done by Eguia et al. assessed the effect of Medicaid expansion in states with coverage in patients with bladder, colorectal, esophageal, lung, bladder, and gastric cancers and found that there was an increase in the number of patients who had access to healthcare and noted a positive effect on the access and utilization of oncologic care [17]. We understand the significance of the implementation of the ACA on the improved access to care utilization; however, the direct correlation between Medicaid expansion and cancer-specific survival (CSS), particularly for early-stage prostate cancer, remains an area warranting comprehensive exploration. This study aims to determine the impact of the implementation of the ACA on survival outcomes, CSS and OS, among men with prostate malignancies.

This article was previously posted to the medRxiv preprint server on October 23, 2024.

## Materials and methods

Data source and study population

This retrospective cohort study utilized data from the Surveillance, Epidemiology, and End Results (SEER) database, focusing on men diagnosed with prostate cancer between 2000 and 2021 [18]. SEER collects cancer incidence and survival data from population-based cancer registries covering approximately 48% of the US population as of 2021 [19]. The study utilized the "Incidence - SEER 18 Registries, Nov 2021 Sub (2000-2021)" database [18]. Inclusion criteria included the following: (1) men diagnosed with prostate malignancies identified using International Classification of Diseases for Oncology, Third Edition (ICD-O-3) codes; (2) patients with complete clinicopathological information and survival data; (3) age at diagnosis between 18 and 64 years; and (4) patients actively followed up. In contrast, exclusion criteria were as follows: (1) patients diagnosed through autopsy or death certificate only or with clinical diagnoses only and (2) patients with missing data on race/ethnicity, household median income, state identification, or years of follow-up. This study involved publicly available data and did not include any identifiable patient information, so institutional review board approval was not required. Informed consent was also not necessary due to the retrospective nature of the study.

Georgia vs. Kentucky

Georgia (the control state) and Kentucky (the treated state) were selected for the study due to their contrasting approaches to healthcare policy, specifically regarding the implementation of the ACA. Kentucky expanded Medicaid under the ACA in 2014, leading to a significant increase in Medicaid/Children's Health Insurance Program (CHIP) enrollment and a reduction in the uninsured rate. In contrast, Georgia did not expand Medicaid, resulting in higher uninsured rates and more restrictive access to healthcare services. The differences in healthcare policy between the two states provide a unique opportunity to assess the impact of the ACA on prostate cancer outcomes, particularly CSS and OS. 

Primary outcomes of interest

The primary outcomes of interest were CSS and OS. CSS was defined as the time from diagnosis to death due to prostate cancer, while OS referred to the time from diagnosis to death from any cause. Both outcomes were assessed over two periods: pre-ACA (2003-2009) and post-ACA (2015-2021). 

Independent variables of interest

The primary independent variables were the implementation of the ACA and Medicaid expansion, categorized into two time periods, pre-ACA (2003-2009) and post-ACA (2015-2021), with a one-year washout period of January 2014-December 2014 to allow for the policy to become fully operational. Kentucky's Medicaid expansion served as the treatment, while Georgia, which did not expand Medicaid, served as the control. The interaction between state and time period (State×ACA) was used to evaluate the differential impact of the ACA on prostate cancer outcomes between the two states. 

Covariates

Covariates included demographic factors such as age at diagnosis (continuous variable), race/ethnicity (categorized as non-Hispanic White people, non-Hispanic Black people, Hispanic people, non-Hispanic Asian/Pacific Islander people, and Native American people), and marital status (categorized as married, widowed, divorced, separated, or unknown). Socioeconomic status was assessed using household median income (>$64,000 and ≤$64,000). Tumor characteristics, including stage at diagnosis (localized, regional, distant) and treatment modalities (prostatectomy, chemotherapy, radiation), were also included as covariates. 

Theoretical model: Andersen's Behavioral Model of Health Services Utilization

This study uses Andersen's Behavioral Model of Health Services Utilization [20,21] to explore the impact of the ACA and Medicaid expansion on prostate cancer mortality, comparing Kentucky (which implemented Medicaid expansion in 2014) and Georgia (which has not). The model categorizes factors into predisposing, enabling, and need factors.

Predisposing Factors

These include demographics such as age (18-45, 45-64 years), race/ethnicity (White, Black, Hispanic, etc.), and marital status (single, married, etc.), which influence healthcare-seeking behavior.

Enabling Factors

These are resources that facilitate access to care, including income (<64K, ≥64K), metropolitan status (rural, small, medium, large metropolitan), and the state of residence (Kentucky vs. Georgia), reflecting the impact of Medicaid expansion.

Need Factors

These include clinical variables such as cancer grade (stage I, stage II, stage III, and stage IV) and the receipt of treatments like surgery, chemotherapy, and radiotherapy, all of which influence prostate cancer outcomes.

Difference-in-differences (DID) specification

The present study used a DID model [22] to estimate the impact of Medicaid expansion on prostate cancer outcomes, OS, overall deaths, and disease stage at presentation, by comparing Kentucky (the treatment group, which implemented Medicaid expansion) to Georgia (the control group, which did not) during the pre-ACA and post-ACA periods. The variable ACA was set to 1 for the post-Medicaid expansion period (2015-2020) and 0 for the pre-expansion period (2003-2009). The variable State was defined as 1 if the observation was from Kentucky (expansion state) and 0 if from Georgia (non-expansion state).

The DID model is specified as follows: $\text{y}=\text{X&beta;}+\text{&beta;1&sdot;ACA}+\text{&beta;2&sdot;State}+\text{&beta;12&sdot; (ACA&times;State)}+\text{u}$ [22]. Here, y represents the prostate cancer outcome of interest (e.g., survival, overall mortality, or disease stage at presentation). X includes covariates such as age, race, marital status, and treatment modalities. β1 captures the difference in outcomes between the pre- and post-ACA periods across both states. β2 captures the baseline difference between Kentucky and Georgia. β12 represents the effect of Medicaid expansion on prostate cancer outcomes. The interaction term ACA×State (i.e., β12) estimates the difference in the change in outcomes between Kentucky (the expansion state) and Georgia (the non-expansion state) from the pre- to the post-ACA period. This term provides the key estimate of the impact of Medicaid expansion.

Missing data

In this study, we addressed missing data by conducting a complete case analysis, which involved excluding observations with missing values on key variables from the analysis. This approach was chosen to ensure the validity and reliability of our findings by focusing on cases with complete information. Prior to the analysis, we assessed the extent and patterns of missing data to understand its potential impact. The proportion of missing data was minimal and unlikely to introduce significant bias. Furthermore, we examined whether the data were missing completely at random (MCAR) by comparing the characteristics of cases with and without missing data. No systematic differences were observed, supporting the appropriateness of a complete case analysis. While this method may reduce sample size and statistical power, it provides a straightforward and transparent approach to handling missing data without introducing additional assumptions required for imputation techniques.

Statistical analysis

Descriptive statistics summarized the characteristics of the study population. Chi-squared tests were used for categorical variables, and appropriate regression models, such as the Cox proportional hazards model, were applied to time-to-event data. Margin plots were generated to visualize the adjusted probabilities of each outcome across states and time periods. Subsequently, the "lincom" command was used to calculate the DID and generate the standard error, p-value, and confidence intervals. The DID analysis was restricted to individuals earning less than $65,000 annually, as this income threshold aligns with Medicaid eligibility criteria. This restriction ensures that the analysis focuses on the population most likely to be impacted by Medicaid expansion under the ACA. Statistical significance was determined using two-tailed tests with an alpha level of 0.05. All analyses were conducted using Stata Statistical Software: Release 16 (2019; StataCorp LLC, College Station, Texas, United States). 

## Results

Baseline study characteristics

In Table 1, the baseline characteristics of individuals with prostate cancer in Georgia and Kentucky were compared across two time periods: pre-ACA (2003-2009) and post-ACA (2015-2021). In the pre-ACA period, the mean age of diagnosis was similar between the two states, with Georgia at 57.3±5.2 years and Kentucky at 57.6±5.0 years. Post-ACA, the mean age slightly increased in both states, with Georgia at 58.1±4.8 years and Kentucky at 58.4±4.7 years. In terms of race and ethnicity, Georgia had a significantly higher proportion of non-Hispanic Black people compared to Kentucky in both periods. Pre-ACA, 37% of patients in Georgia were non-Hispanic Black people, compared to only 10.5% in Kentucky. Post-ACA, the proportion of non-Hispanic Black people in Georgia further increased to 47.7%, while in Kentucky, it remained relatively low at 14.2%.

**Table 1:** Demographic, clinical, and treatment characteristics of individuals with prostate cancer in Georgia and Kentucky before and after Medicaid expansion in Kentucky in 2014 The table summarizes the key demographic, clinical, and treatment differences among prostate cancer patients in Georgia and Kentucky before (2003-2009) and after (2015-2021) Medicaid expansion in Kentucky. "Localized" refers to cancer confined to the prostate; "regional" indicates cancer that has spread to nearby lymph nodes or tissues; and "distant metastasis" refers to cancer that has spread to distant organs or parts of the body. ACA: Affordable Care Act

Variables	Pre-ACA (N=37,810)			Post-ACA (N=30,412)		
	Georgia (N=26,772)	Kentucky (N=11,038)	P-value	Georgia (N=48,910)	Kentucky (N=19,312)	P-value
Age (years; mean±SD)	57.3±5.2	57.6±5.0	<0.01	58.1±4.8	58.4±4.7	<0.01
Age (years)						
18-44	567 (2.1%)	163 (1.5%)	<0.01	251 (1.1%)	72 (0.9%)	0.046
45-64	26,205 (97.9%)	10,875 (98.5%)		21,887 (98.9%)	8,202 (99.1%)	
Race/ethnicity						
White person	16,158 (60.4%)	9,698 (87.9%)	<0.01	10,366 (46.8%)	6,679 (80.7%)	<0.01
Black person	9,895 (37%)	1,163 (10.5%)		10,566 (47.7%)	1,171 (14.2%)	
Hispanic person	453 (1.7%)	57 (0.5%)		740 (3.3%)	80 (1%)	
Other	266 (1%)	120 (1.1%)		466 (2.1%)	344 (4.2%)	
Income						
<65K	12,679 (47.4%)	9,432 (85.5%)	<0.001	9,739 (44%)	7,008 (84.7%)	<0.001
≥65K	14,093 (52.6%)	1,606 (14.6%)		12,399 (56%)	1,266 (15.3%)	
Marital status						
Single	2,944 (11%)	754 (6.8%)	<0.001	3,465 (15.7%)	968 (11.8%)	<0.01
Married	18,772 (70.1%)	7,358 (66.7%)		12,862 (58.3%)	4,883 (59.3%)	
Widowed	507 (1.9%)	196 (1.8%)		331 (1.5%)	162 (2%)	
Divorced	2,277 (8.5%)	997 (9%)		1,566 (7.1%)	845 (10.3%)	
Separated	273 (1%)	56 (0.5%)		197 (0.9%)	46 (0.6%)	
Unknown	1,988 (7.5%)	1,677 (15.2%)		3,649 (16.5%)	1,334 (16.2%)	
Metropolitan status						
Rural areas not adjacent to metropolitan areas	1,081 (4%)	2,464 (22.3%)	<0.001	930 (4.2%)	1,900 (23%)	<0.001
Rural areas adjacent to metropolitan areas	3,648 (13.6%)	1,809 (16.4%)		2,437 (11%)	1,273 (15.4%)	
Small metropolitan areas	4,037 (15.1%)	925 (8.4%)		3,020 (13.6%)	793 (9.6%)	
Medium metropolitan areas	3,005 (11.2%)	1,704 (15.4%)		2,459 (11.1%)	1,262 (15.3%)	
Large metropolitan areas	15,001 (56%)	4,136 (37.5%)		13,292 (60%)	3,046 (36.8%)	
Stage at presentation						
Localized	15,383 (85.8%)	5,731 (80.5%)	<0.01	6,855 (77.2%)	2,395 (68.5%)	<0.01
Regional	1,802 (10.1%)	1,081 (15.2%0		1,237 (13.9%)	557 (15.9%)	
Distant	480 (2.7%)	196 (2.8%)		463 (5.2%)	160 (4.6%)	
Unknown	271 (1.5%)	109 (1.5%)		328 (3.7%)	384 (11%)	
Surgery, n (%)						
Yes	14,217 (54%)	3,635 (35.7%)	<0.001	13,867 (63%)	4,088 (54.8%)	<0.01
None/unknown	12,135 (46.1%)	6,551 (64.3%)		8,143 (37%)	3,369 (45.2%)	
Chemotherapy, n (%)						
Yes	191 (0.7%)	69 (0.6%)	0.345	445 (2%)	191 (2.3%)	0.106
None/unknown	26,581 (99.2%)	10,969 (99.4%)		21,693 (98%)	8,083 (97.7%)	
Radiotherapy, n (%)						
Yes	7,994 (34.2%)	1,908 (19.5%)	<0.01	5,210 (25.5%)	2,197 (27.6%)	<0.01
None/unknown	15,390 (65.8%)	7,898 (80.5%)		15,242 (74.5%)	5,766 (72.4%)	

In terms of tumor stage at diagnosis, the proportion of localized cases decreased significantly in both states post-ACA. In Georgia, localized cases dropped from 85.8% pre-ACA to 77.2% post-ACA, while in Kentucky they decreased from 80.5% to 68.5%. Additionally, the incidence of distant-stage prostate cancer increased in both states post-ACA, rising from 2.7% to 5.2% in Georgia and from 2.8% to 4.6% in Kentucky. Metropolitan status also revealed significant differences, with a higher proportion of patients in Kentucky residing in rural areas compared to Georgia during both periods. For example, in the post-ACA period, 23% of patients in Kentucky lived in rural areas not adjacent to metropolitan areas, compared to only 4.2% in Georgia (Table 1).

Factors associated with CSS

Table 2 reveals the factors associated with CSS among individuals with prostate cancer, comparing Georgia and Kentucky across pre-ACA and post-ACA periods. The post-2015 period was associated with improved survival outcomes, with a hazard ratio (HR) of 0.78 (95% CI: 0.68-0.89), indicating a significant reduction in the risk of cancer-specific mortality. Additionally, the interaction between states and the post-Medicaid expansion period revealed that individuals in Kentucky had a substantial reduction in mortality post-expansion period (HR=0.71; 95% CI: 0.56-0.90), suggesting a beneficial effect of the policy in reducing cancer-specific mortality in this state.

**Table 2:** Cox proportional hazards model assessing factors associated with cancer-specific mortality among individuals with prostate cancer ACA: Affordable Care Act; HR: hazard ratio; SE: standard error; CI: confidence interval

Variables	HR	SE	z	P>z	95% CI
State					
Kentucky (ref. Georgia)	1.112	0.068	1.750	0.080	0.987-1.253
ACA					
Post-ACA (ref. pre-ACA)	0.780	0.054	-3.610	0.000	0.682-0.893
State×ACA					
Kentucky×post-ACA	0.711	0.087	-2.770	0.006	0.559-0.905
Age (years)					
18-44	Reference				
45-64	1.440	0.266	1.980	0.048	1.003-2.068
Race/ethnicity					
Whites	Reference				
Blacks	1.103	0.055	1.970	0.049	1.000-1.215
Hispanics	0.862	0.154	-0.830	0.406	0.608-1.223
Asians/Pacific Islanders	1.229	0.374	0.680	0.499	0.676-2.233
Native Americans	0.465	0.465	-0.770	0.444	0.065-3.308
Unknown	0.390	0.227	-1.620	0.106	0.125-1.220
Marital status					
Single	Reference				
Married	0.653	0.040	-6.980	0.000	0.580-0.736
Widowed	1.065	0.148	0.450	0.652	0.810-1.399
Divorced	0.834	0.066	-2.310	0.021	0.714-0.973
Separated	0.835	0.154	-0.980	0.330	0.581-1.200
Unknown	0.863	0.078	-1.630	0.102	0.723-1.030
Metropolitan status					
Rural areas not adjacent to metropolitan areas	Reference				
Rural areas adjacent to metropolitan areas	0.915	0.077	-1.050	0.294	0.775-1.080
Small metropolitan areas	0.752	0.068	-3.140	0.002	0.629-0.898
Medium metropolitan areas	0.816	0.073	-2.270	0.023	0.684-0.973
Large metropolitan areas	0.738	0.062	-3.630	0.000	0.626-0.870
Grade					
Localized	Reference				
Regional	1.269	0.268	1.130	0.258	0.839-1.919
Distant	4.357	0.981	6.540	0.000	2.803-6.773
Disease stage at presentation					
Stage I	Reference				
Stage II	3.203	1.152	3.240	0.001	1.583-6.482
Stage III	10.432	4.363	5.610	0.000	4.596-23.677
Stage IV	26.988	11.333	7.850	0.000	11.851-61.461
Household median income					
Income ≥64K (ref. <64K)	0.935	0.055	-1.140	0.253	0.832-1.049
Treatment modality					
Prostatectomy	0.378	0.025	-14.840	0.000	0.332-0.430
Chemotherapy	1.622	0.127	6.180	0.000	1.391-1.891
Radiation	1.204	0.060	3.740	0.000	1.092-1.327

Other covariates also played a critical role in the CSS. Older age (45-64 years) was associated with increased mortality (HR=1.44; 95% CI: 1.00-2.07), while being married was protective (HR=0.65; 95% CI: 0.58-0.74). Racial disparities were evident, with non-Hispanic Black people having a higher risk of mortality (HR=1.10; 95% CI: 1.00-1.22) compared to non-Hispanic White people. Socioeconomic factors, such as living in large metropolitan areas, were associated with improved survival (HR=0.74; 95% CI: 0.63-0.87). Advanced cancer stages were strongly associated with worse outcomes, with stage IV having the highest mortality risk (HR=26.99; 95% CI: 11.85-61.46). Additionally, treatment factors such as receiving a prostatectomy significantly reduced the risk of mortality (HR=0.38; 95% CI: 0.33-0.43), while chemotherapy and radiation were associated with increased mortality risk (HR=1.62; 95% CI: 1.39-1.89; HR=1.20; 95% CI: 1.09-1.33, respectively) (Table 2).

Factors associated with OS

Table 3 reports the factors associated with the OS among individuals with prostate cancers in both states and in both time periods. The post-2015 period was associated with improved OS, with an HR of 0.90 (95% CI: 0.82-0.99), indicating a significant reduction in the risk of overall mortality during the period. The interaction between state and post-Medicaid expansion period showed that individuals in Kentucky had a reduction in mortality post-ACA (HR=0.87; 95% CI: 0.75-1.01), although this effect was marginally significant (p=0.065).

**Table 3:** Cox proportional hazards model assessing factors associated with overall deaths among individuals with prostate cancer ACA: Affordable Care Act; HR: hazard ratio; SE: standard error; CI: confidence interval; NHAPI: non-Hispanic Asian/Pacific Islander

Variables	HR	SE	z	P>z	95% CI
State					
Kentucky (ref. Georgia)	1.181	0.039	5.050	0.000	1.107-1.259
ACA					
Post-ACA (ref. pre-ACA)	0.901	0.042	-2.260	0.024	0.823-0.986
State×ACA					
Kentucky×post-ACA	0.868	0.067	-1.850	0.065	0.747-1.009
Age (years)					
18-44	Reference				
45-64	2.175	0.283	5.980	0.000	1.686-2.806
Race/ethnicity					
Whites	Reference				
Blacks	1.103	0.031	3.480	0.001	1.044-1.166
Hispanics	0.983	0.103	-0.170	0.867	0.801-1.206
NHAPI	0.741	0.162	-1.370	0.171	0.482-1.139
Native Americans	0.436	0.309	-1.170	0.241	0.109-1.747
Unknown	0.229	0.087	-3.880	0.000	0.109-0.482
Marital status					
Single	Reference				
Married	0.649	0.023	-11.960	0.000	0.605-0.697
Widowed	1.126	0.091	1.470	0.141	0.961-1.319
Divorced	0.994	0.046	-0.130	0.895	0.908-1.088
Separated	1.060	0.112	0.550	0.581	0.861-1.305
Unknown	0.751	0.040	-5.430	0.000	0.678-0.833
Metropolitan status					
Rural areas not adjacent to metropolitan areas	Reference				
Rural areas adjacent to metropolitan areas	0.964	0.045	-0.770	0.441	0.880-1.058
Small metropolitan areas	0.819	0.041	-3.990	0.000	0.743-0.904
Medium metropolitan areas	0.776	0.039	-5.060	0.000	0.703-0.856
Large metropolitan areas	0.713	0.033	-7.340	0.000	0.652-0.781
Grade					
Localized	Reference				
Regional	1.133	0.099	1.430	0.154	0.954-1.345
Distant	3.482	0.387	11.230	0.000	2.801-4.329
Disease stage at presentation					
Stage I	Reference				
Stage II	1.449	0.168	3.200	0.001	1.155-1.819
Stage III	2.135	0.318	5.100	0.000	1.595-2.858
Stage IV	4.047	0.625	9.060	0.000	2.991-5.477
Household median income					
Income ≥64K (ref. <64K)	0.878	0.030	-3.840	0.000	0.822-0.938
Treatment modality					
Prostatectomy	0.473	0.017	-21.050	0.000	0.441-0.507
Chemotherapy	1.421	0.097	5.130	0.000	1.243-1.625
Radiation	1.017	0.032	0.530	0.598	0.956-1.082

Other covariates also played a crucial role in OS. Age between 45 and 64 years was significantly associated with higher mortality (HR=2.18; 95% CI: 1.69-2.81), while being married was also protective (HR=0.65; 95% CI: 0.61-0.70). Racial disparities were evident, with non-Hispanic Black people having a higher risk of mortality (HR=1.10; 95% CI: 1.04-1.17) compared to non-Hispanic White people. Socioeconomic factors, such as living in large metropolitan areas, were associated with improved survival (HR=0.71; 95% CI: 0.65-0.78). Advanced cancer stages were strongly associated with worse outcomes, with stage IV having the highest mortality risk (HR=4.05; 95% CI: 2.99-5.48). Additionally, treatment factors such as receiving a prostatectomy significantly reduced the risk of mortality (HR=0.47; 95% CI: 0.44-0.51), while chemotherapy was associated with increased mortality risk (HR=1.42; 95% CI: 1.24-1.62) (Table 3).

Predicted probabilities (CSS)

Table 4 reveals the predicted probabilities of the hazards of death for prostate cancer between Georgia and Kentucky in the pre-ACA and post-ACA across different races/ethnicities. In both states and across all races and ethnicities, the post-2015 period was associated with an improvement in CSS.

**Table 4:** Predicted probabilities of cancer-specific mortality among individuals with prostate cancer This table compares the predicted hazards of prostate cancer-specific mortality in Georgia and Kentucky before (pre-ACA) and after (post-ACA) the implementation of the ACA. The results indicate a decline in mortality hazards, reflecting improved cancer-specific survival in both states during the post-ACA period. This trend is consistent across different racial and ethnic groups. ACA: Affordable Care Act; NHAPI: non-Hispanic Asian/Pacific Islander

Variables	All	Whites	Blacks	Hispanics	NHAPI	Native Americans
Georgia						
Pre-ACA	1.06 (0.81-1.31)	1.02 (0.78-1.26)	1.13 (0.86-1.40)	1.00 (0.69-1.32)	0.76 (0.39-1.13)	0.45 (-0.18 to 1.07)
Post-ACA	0.95 (0.70-1.21)	0.92 (0.67-1.17)	1.02 (0.74-1.29)	0.91 (0.60-1.21)	0.68 (0.34-1.03)	0.40 (-0.17 to 0.97)
Kentucky						
Pre-ACA	1.25 (0.94-1.56)	1.21 (0.91-1.51)	1.33 (0.99-1.67)	1.19 (0.81-1.57)	0.89 (0.45-1.34)	0.53 (-0.21 to 1.27)
Post-ACA	0.98 (0.70-1.26)	0.94 (0.67-1.22)	1.04 (0.73-1.35)	0.93 (0.60-1.25)	0.70 (0.34-1.06)	0.41 (-0.17 to 0.99)

The post-2015 period in Georgia experienced a reduction in the probability of death from 1.06 (95% CI: 0.81-1.31) pre-ACA to 0.95 (95% CI: 0.70-1.21) post-ACA. Similarly, in Kentucky, the probability of death decreased from 1.25 (95% CI: 0.94-1.56) pre-ACA to 0.98 (95% CI: 0.70-1.26) post-ACA. Among non-Hispanic White people, a similar trend was observed, with the pre-ACA probability decreasing from 1.02 (95% CI: 0.78-1.26) to 0.92 (95% CI: 0.67-1.17) in Georgia and from 1.21 (95% CI: 0.91-1.51) to 0.94 (95% CI: 0.67-1.22) in Kentucky.

For non-Hispanic Black people, the predicted probability of death post-ACA decreased from 1.13 (95% CI: 0.86-1.40) to 1.02 (95% CI: 0.74-1.29) in Georgia and from 1.33 (95% CI: 0.99-1.67) to 1.04 (95% CI: 0.73-1.35) in Kentucky. Hispanic people also experienced a decrease in the probability of death, particularly in Kentucky, where the post-ACA period saw a reduction from 1.00 (95% CI: 0.69-1.32) to 0.91 (95% CI: 0.60-1.21). This pattern was also seen among non-Hispanic Asian/Pacific Islander people and Native American people (Table 4).

Predicted probabilities (OS)

Table 5 shows the predicted probabilities of overall deaths between Georgia and Kentucky in the pre-ACA and post-ACA periods across racial and ethnic groups. Specifically, the probability of death decreased from 5.41 (95% CI: 1.55-9.28) in the pre-ACA period to 4.22 (95% CI: 1.10-7.35) in the post-ACA period. In Georgia, there was a reduction from 6.02 (95% CI: 1.63-10.41) to 3.34 (95% CI: 0.80-5.88) in Kentucky. Among non-Hispanic White people, the probabilities followed a similar trend, with the post-ACA period showing reduced risks compared to the pre-ACA period, such as in Kentucky, where the probability decreased from 5.79 (95% CI: 1.58-10.00) to 3.21 (95% CI: 0.78-5.65). Non-Hispanic Black people also experienced a reduction in the predicted probabilities of death post-ACA, with a decrease from 6.39 (95% CI: 1.69-11.09) to 3.54 (95% CI: 0.82-6.27) in Kentucky. Hispanic people, non-Hispanic Asian/Pacific Islander people, and Native American people also showed a reduction in the post-ACA period across both states (Table 5).

**Table 5:** Predicted probabilities of overall mortality among individuals with prostate cancer This table compares the predicted hazards of prostate cancer-specific mortality in Georgia and Kentucky before (pre-ACA) and after (post-ACA) the implementation of the ACA. The results indicate a decline in mortality hazards, reflecting improved cancer-specific survival in both states during the post-ACA period. This trend is consistent across different racial and ethnic groups. ACA: Affordable Care Act; NHAPI: non-Hispanic Asian/Pacific Islander

Variables	All	Whites	Blacks	Hispanics	NHAPI	Native Americans
Georgia						
Pre-ACA	5.41 (1.55-9.28)	5.21 (1.49-8.93)	5.74 (1.61-9.88)	4.49 (0.94-8.04)	6.40 (0.44-12.36)	2.42 (-2.63 to 7.47)
Post-ACA	4.22 (1.10-7.35)	4.07 (1.06-7.07)	4.48 (1.15-7.82)	3.51 (0.67-6.35)	5.00 (0.25-9.75)	1.89 (-2.06 to 5.84)
Kentucky						
Pre-ACA	6.02 (1.63-10.41)	5.79 (1.58-10.00)	6.39 (1.69-11.09)	5.00 (0.98-9.01)	7.12 (0.41-13.83)	2.69 (-2.94 to 9.32)
Post-ACA	3.34 (0.80-5.88)	3.21 (0.78-5.65)	3.54 (0.82-6.27)	2.77 (0.47-5.08)	3.95 (0.13-7.77)	1.49 (-1.64 to 4.63)

DID for CSS

Table 6 reports the DID output on the change in the hazards of death for CSS among individuals with prostate cancer. Overall, the coefficient for all individuals was -1.49 (95% CI: -3.00 to 0.02; p=0.05), indicating a marginally significant reduction in the hazard of death in Kentucky over Georgia. When broken down by race/ethnicity, non-Hispanic White people showed a similar reduction (Coef.=-1.44; 95% CI: -2.89 to 0.01; p=0.052), as did non-Hispanic Black people (Coef.=-1.58; 95% CI: -3.19 to 0.02; p=0.054), both nearing statistical significance. Hispanic people also experienced a reduction in hazards (Coef.=-1.24; 95% CI: -2.55 to 0.08; p=0.065), though the result was slightly less significant. The reduction was more pronounced for non-Hispanic Asian/Pacific Islander people (Coef.=-1.76; 95% CI: -3.84 to 0.31; p=0.096), though it did not reach statistical significance. Native Americans showed the least reduction in hazard, with a coefficient of -0.67 (95% CI: -2.14 to 0.81; p=0.376), indicating no significant change (Table 6).

**Table 6:** Difference-in-difference output for cancer-specific mortality by race/ethnicity This table compares changes in prostate cancer-specific mortality hazards between Kentucky and Georgia across pre- and post-ACA periods. The results show a non-significant reduction in mortality hazards in Kentucky relative to Georgia post-ACA across all racial and ethnic groups. ACA: Affordable Care Act; SE: standard error; CI: confidence interval

	Coef.	SE	Z	P>z	95% CI
All	-1.492	0.770	-1.940	0.053	-3.000 to 0.017
Whites	-1.436	0.740	-1.940	0.052	-2.885 to 0.014
Blacks	-1.583	0.820	-1.930	0.054	-3.191 to 0.025
Hispanics	-1.238	0.671	-1.840	0.065	-2.554 to 0.078
Asians/Pacific Islanders	-1.764	1.059	-1.670	0.096	-3.839 to 0.311
Native Americans	-0.667	0.753	-0.890	0.376	-2.142 to 0.808

DID for OS

Table 7 reveals the DID results on the change in the hazard of death for OS among individuals with prostate cancers. There was a substantive reduction in mortality risk across most racial and ethnic groups post-Medicaid expansion in Kentucky compared to Georgia post-ACA implementation. The analysis showed a significant decrease in the hazard of death, with a coefficient of -0.17 (95% CI: -0.33 to -0.01; p=0.039). This trend was consistent across non-Hispanic White people (Coef.=-0.16; 95% CI: -0.32 to -0.01; p=0.039) and non-Hispanic Black people (Coef.=-0.18; 95% CI: -0.35 to -0.01; p=0.039), both of which experienced significant reductions in mortality risk. Hispanic people also experienced a significant reduction in hazards (Coef.=-0.16; 95% CI: -0.31 to -0.01; p=0.043). Non-Hispanic Asian/Pacific Islander people showed a similar, though marginally significant, reduction (Coef.=-0.12; 95% CI: -0.24 to 0.00; p=0.060). Native Americans had the smallest reduction, with a coefficient of -0.07 (95% CI: -0.19 to 0.05; p=0.245), indicating no significant change in OS (Table 7).

**Table 7:** Difference-in difference output for overall deaths by race/ethnicity This table compares changes in prostate cancer-specific mortality hazards between Kentucky and Georgia across pre- and post-ACA periods. The results show a significant reduction in mortality hazards in Kentucky relative to Georgia across all racial and ethnic groups, with the highest improvement observed among Black individuals. ACA: Affordable Care Act; SE: standard error; CI: confidence interval

	Coef.	SE	z	P>z	95% CI
All	-0.168	0.081	-2.070	0.039	-0.326 to -0.009
Whites	-0.162	0.078	-2.070	0.039	-0.315 to -0.008
Blacks	-0.179	0.086	-2.070	0.039	-0.348 to -0.009
Hispanics	-0.159	0.079	-2.030	0.043	-0.313 to -0.005
Asians/Pacific Islanders	-0.120	0.064	-1.880	0.060	-0.245 to 0.005
Native Americans	-0.071	0.061	-1.160	0.245	-0.190 to 0.048

## Discussion

This study demonstrates an improvement in survival among individuals diagnosed with prostate cancer in the post-2015 period, observed across both Kentucky and Georgia, regardless of Medicaid expansion status. However, no significant difference in CSS was detected between the two states following Medicaid expansion. Also, we observed a significant improvement in OS in Kentucky, which implemented the ACA and its Medicaid expansion in 2014, compared to non-expansion in Georgia. There was a significant 16.8 percentage point decrease in overall mortality risk among individuals with prostate cancer in Kentucky relative to Georgia, with the reduction ranging from -32.6% to -9%. This improvement in OS was consistent across different racial/ethnic groups and was particularly significant among Black individuals, who experienced a 17.9 percentage point drop in the risk of overall deaths compared to their counterparts in Georgia. Other significant predictors of improved CSS and OS included younger age, White race, higher household median income, married status, and residence in metropolitan areas. Undergoing prostatectomy was significantly associated with improved survival, while radiation therapy did not show a significant effect. In contrast, chemotherapy was linked to worse CSS and OS, potentially due to a higher disease burden among individuals receiving this treatment modality.

The improvement in survival among individuals diagnosed with prostate cancer in recent years is likely multifactorial [23-25]. Advances in early detection and screening methods, particularly the widespread adoption of prostate-specific antigen (PSA) testing, have facilitated diagnosis at earlier, more treatable stages [26-28]. Additionally, innovations in treatment modalities, including the development of more effective surgical techniques, radiotherapy, androgen deprivation therapy, and novel targeted therapies, have significantly enhanced disease management [24,29-31]. Increased access to multidisciplinary care, improved supportive therapies, and heightened awareness of prostate cancer through public health campaigns have also contributed to better outcomes [32-34]. Furthermore, there have been an overall improvement in healthcare delivery systems and a greater emphasis on personalized treatment approaches tailored to patients' genetic and molecular profiles, which may account for the observed survival gains [35,36].

When comparing the change in prostate cancer-specific mortality between Kentucky and Georgia, there were no statistically significant differences, although the results indicated a trend towards a greater decline in Kentucky. This suggests that while there may be a modest reduction in prostate cancer-specific deaths following the implementation of the ACA and Medicaid expansion in Kentucky, the findings do not provide robust evidence of a substantive improvement in outcomes relative to Georgia.

The absence of a substantive impact of Medicaid expansion on prostate CSS in Kentucky compared to Georgia may be attributed to several factors. Prostate cancer is generally a slow-growing malignancy with a favorable prognosis, and the benefits of improved healthcare access may take years to manifest in survival outcomes [37,38]. The indolent nature of the disease, coupled with high baseline survival rates, could dilute the measurable effects of policy changes within the study timeframe [39,40]. Moreover, while we observed a trend towards a greater decline in prostate cancer-specific mortality in Kentucky, the lack of statistical significance suggests that the improvement may be influenced by other concurrent healthcare advancements or socioeconomic factors not directly related to Medicaid expansion [10,41]. These nuances indicate that longer follow-up periods or studies focusing on more aggressive cancers might be needed to detect the full impact of Medicaid expansion on cancer-specific outcomes [42].

However, Kentucky, a Medicaid expansion state, demonstrated a significant 16.8% reduction in overall mortality risk compared to Georgia, a non-expansion state. This is particularly significant because overall mortality, unlike cancer-specific mortality, may more comprehensively capture the broader benefits of Medicaid expansion, such as improved access to preventive care, management of comorbidities, and timely interventions for acute conditions. Given the generally indolent nature of prostate cancer, the impact of Medicaid expansion on prostate cancer-specific deaths may take longer to manifest, whereas the significant reduction in all-cause mortality in Kentucky suggests that enhanced healthcare access had an immediate and measurable effect on broader health outcomes.

This finding is important in light of the fact that no statistically significant change in overall mortality was observed across the study period in either state, emphasizing that the mortality reduction in Kentucky may be directly attributable to increased healthcare access following the implementation of the ACA and its Medicaid expansion component. These results highlight the potential of Medicaid expansion to reduce health disparities and improve survival, particularly in populations that were previously uninsured or underinsured, reinforcing the role of policy-driven healthcare reforms in enhancing population health outcomes [22,43,44].

Interestingly, individuals who received chemotherapy experienced a significantly higher hazard of both cancer-specific and overall mortality. This may be explained by a higher disease burden and more advanced cancer stage at the time of treatment, as chemotherapy is often reserved for high-risk or metastatic cases [45-47]. Additionally, the observed mortality risk may reflect an imbalance in sociodemographic and clinical factors, as patients receiving chemotherapy were more likely to belong to vulnerable groups such as low-income families, unmarried or single individuals, and racial minority populations that often have limited access to comprehensive healthcare and may present with more aggressive disease [48,49]. Moreover, disparities in treatment adherence and supportive care in these groups could further contribute to poorer outcomes, underscoring the need for tailored interventions to address these inequities and improve survival among chemotherapy-treated patients [50-52].

This study highlights persistent disparities in cancer outcomes, with Black individuals, those from the lowest-income households, single and unmarried individuals, and residents of rural areas facing the highest risk of both cancer-specific and overall mortality. These disparities may be driven by a combination of factors, including delayed diagnosis, limited access to high-quality care, and a greater burden of comorbidities in these populations. Additionally, socioeconomic barriers and geographical challenges often result in reduced access to advanced treatments and follow-up care, further exacerbating survival disparities.

Study limitations

This study has several strengths, including the use of the SEER registry, a high-quality, population-based cancer database that provides comprehensive clinical and demographic information, allowing for robust analysis of cancer outcomes. A key strength is our approach of comparing two states, Kentucky and Georgia, rather than broadly categorizing states into Medicaid expansion versus non-expansion groups. This approach reduces potential bias stemming from heterogeneity in Medicaid implementation timelines and healthcare infrastructure across states, enhancing the specificity of our findings. Additionally, the use of a DID approach strengthens causal inference by accounting for underlying time trends, providing a more accurate estimate of Medicaid expansion's impact on survival outcomes.

Our study has several limitations that should be acknowledged. First, we employed a complete case analysis, excluding patients with missing data, which may introduce selection bias and limit the generalizability of our findings. Additionally, exclusions due to missing data may not be random, potentially impacting the validity of our results. Second, the absence of critical variables, such as comorbidity information, baseline insurance status, and patient-reported outcomes, limits our ability to control for pre-existing health conditions and other unmeasured confounders, which could affect survival outcomes. Third, while we focused on Kentucky and Georgia to evaluate the impact of Medicaid expansion, differences in healthcare quality, access to advanced therapies, and variations in healthcare delivery systems between these states, unrelated to Medicaid expansion, may have influenced our results and were not fully accounted for.

Fourth, the short follow-up period restricts our ability to assess the long-term effects of Medicaid expansion, particularly for slow-progressing cancers like prostate cancer. Addressing this limitation in future research may involve including cancers with shorter survival times or incorporating longer follow-up periods to better capture the effects of policy changes. Fifth, while our analysis adjusted for metropolitan status and included stratifications by race and income, residual confounding remains a concern. Unobserved variables, such as patient quality of life and differences in healthcare utilization, may still influence the observed outcomes despite our statistical adjustments.

Lastly, the use of the publicly available SEER database is both a strength and a limitation. While it facilitates replication and validation of our findings, the lack of detailed information on healthcare access and patient-reported outcomes reduces the depth of causal inference. Future research should consider integrating data from additional states to enhance generalizability, adding patient-reported measures to capture healthcare experiences, and examining less-successful cancers to provide more real-time data on CSS. These steps will improve our understanding of the long-term impact of Medicaid expansion on health outcomes.

## Conclusions

While our analysis did not identify a significant difference in prostate cancer-specific mortality or survival between Kentucky and Georgia, Kentucky's Medicaid expansion was associated with a significant reduction in all-cause mortality, indicating an improvement in OS. This finding underscores the broader, system-wide impact of Medicaid expansion on health outcomes beyond cancer-specific measures, potentially driven by improved healthcare access. However, it is important to interpret these results cautiously, as the improvements in OS may reflect broader systemic differences unrelated to Medicaid expansion, including variations in healthcare quality and other unmeasured factors.

Significant disparities also persist, particularly among Black individuals, low-income households, rural residents, and unmarried individuals, who experience poorer outcomes in both CSS and OS. These findings highlight the need for targeted interventions to address these inequities and ensure that expanded healthcare coverage reaches the most vulnerable populations.

Future research should aim to disentangle the effects of Medicaid expansion from other systemic healthcare differences, incorporate longer follow-up periods, and include additional states to improve generalizability. Investigating cancers with shorter survival times, integrating patient-reported data, and examining healthcare quality indicators would provide more comprehensive insights into the policy's impact on healthcare equity and cancer-related outcomes.
